# Multinucleated stromal giant cells in the gastroesophageal junctional and gastric mucosa: a retrospective study

**DOI:** 10.1186/s13000-019-0860-y

**Published:** 2019-07-27

**Authors:** Taha Sachak, Wendy L. Frankel, Christina A. Arnold, Wei Chen

**Affiliations:** Department of Pathology, The Ohio State University Wexner Medical Center, S301 Rhodes Hall, 450 W. 10th Ave, Columbus, OH 43210 USA

**Keywords:** Multinucleated stromal giant cells, Reactive changes, Gastroesophageal reflux disease, Chemical gastropathy

## Abstract

**Background:**

Atypical multinucleated stromal giant cells (MSGCs) are occasionally encountered in the esophagogastric mucosa. This study aims to investigate the origin and clinical association of MSGCs in the upper gastrointestinal tract.

**Methods:**

Three hundred sixty-one contiguous biopsies and 1 resection specimen from the stomach and gastroesophageal junction (GEJ) were identified from archives for morphologic and immunohistochemical studies.

**Results:**

MSGCs were identified in 22 cases (6%: 7 gastric, 15 GEJ). Patients’ average age was 53 years. There was no sex predilection. 77% cases had only 1 or 2 MSGCs per 10 high power fields. MSGCs were located in the lamina propria of the gastric or GEJ mucosa, with an accentuation in the subepithelial zone. The median number of nuclei in a MSGC was 5 (ranging from 3 to 16). The nuclei were touching/overlapping, often arranged into “wreath”, “caterpillar”, or “morula” configurations. MSGCs expressed smooth muscle actin, desmin, while negative for cytokeratin AE1/3, CD68, S100, chromogranin, and CD117. The most common clinical history was epigastric pain, gastroesophageal reflux, and Barrett esophagus. The most common associated pathologic diagnoses included reactive (chemical) gastropathy (71% gastric biopsies) and gastroesophageal reflux (73% GEJ specimens).

**Conclusions:**

MSGCs in the esophagogastric mucosa show smooth muscle/myofibroblast differentiation by immunohistochemistry and likely represent a reactive/reparative stromal reaction associated with gastroesophageal reflux and reactive (chemical) gastropathy.

## Introduction

Reactive/reparative changes of the gastrointestinal tract are commonly observed in the daily practice of surgical pathology, secondary to infection, inflammation, foreign body, and others. Multinucleated cells are often seen in this setting. One of the most well-known multinucleated giant cells is the Langhans giant cell, the hallmark of tuberculosis and sarcoidosis. Langhans giant cells are formed by the fusion of multiple macrophages, and feature peripherally placed nuclei surrounding central ample cytoplasm that may contain lipid-rich material. Similarly, foreign-body giant cells are also derived from fused macrophages that engulf endogenous (such as cholesterol, keratin, fat) or exogenous substances (such as suture, talc, fungus, food particles). Squamous epithelium and hepatocytes can also become multi-nucleated in reactive conditions or secondary to viral infection. Benign multinucleated stromal giant cells are well known to exist at various sites, most commonly in the bladder, lower female genital tract, skin, anus, nose, breast, and testis [1–10]. While multinucleated stromal giant cells (MSGCs) have been described previously in the lower gastrointestinal tract [1–3, 6], they have not been characterized in the upper gastrointestinal tract, to the best of our knowledge.

In this study, our goal is to identify the origin and significance of MSGCs in the esophagogastric mucosa, by histomorphology and immunohistochemistry. Awareness and better understanding of MSGCs may be helpful for general surgical pathologists who have limited experience with such cells in the upper gastrointestinal mucosa.

## Materials and methods

This study is approved by Institutional Review Board of The Ohio State University Wexner Medical Center. 361 consecutive biopsies and 1 resection specimen from stomach and GEJ were identified from institutional Archives in January 2016. Pertinent clinical data (patient age, sex, signs and symptoms, clinical indication for endoscopy) and pathologic diagnoses were collected from electronic medical records. Histologic evaluation included the following parameters: location of MSGCs within the upper GI mucosa, morphologic features of MSGCs, the number of MSGCs per 10 high power fields, and pathologic changes of the background mucosa. 17 MSGCs cases (6 stomach; 11 GEJ) were immunostained for the following antibodies: smooth muscle actin (SMA, Dako Clone 1A4, dilution 1:600), desmin (Dako Clone D33, dilution 1:200), CD117 (Dako Rabbit Polyclonal, dilution 1:400), S100 (Dako Rabbit Polyclonal, dilution 1:4000), cytokeratin AE1/3 (Dako Clone AE1/AE3, dilution 1:300), chromogranin (Cell Marque Clone LK2H10, dilution 1:300), and CD68 (Dako Clone KP-1, dilution 1:700). Two sections were cut on each case for each immunostain, to increase the yield of detecting the MSGCs.

Peroxidase immunohistochemical staining was performed and described briefly as follows: Paraffin embedded tissue was cut at 4 μm and placed on positively charged slides. The slides were deparaffinized, rehydrated, and then were placed in a 3% hydrogen peroxide solution in water for 5 min to block for endogenous peroxidase. Antigen retrieval was performed by Heat-Induced Epitope Retrieval (HIER), in which the slides were placed in a 1X solution of Target Retrieval Solution (Dako, S1699) for 25 min at 96 °C using a vegetable steamer (Black & Decker) and cooled for 15 min in solution. Slides were then incubated on a Dako Autostainer Immunostaining System at room temperature. The primary antibodies were diluted with an antibody diluent (Dako, S0809). Antibodies were incubated for 60 min. Staining was visualized with the DAB+ chromogen (Dako, K3468) using a 5-min development. Slides were then counterstained in Richard Allen hematoxylin, dehydrated through graded ethanol solutions, cleared in xylene and coverslipped.

All cases were reviewed by 2 pathologists (T.S. and W.C.); additional opinions were sought from W.L.F. and C.A.A. in a subset of cases. Statistical analysis was performed using Fisher’s exact test.

## Results

MSGCs were identified in 22 out of 362 gastroesophageal specimens (6%). 7 cases were gastric endoscopic biopsies, 14 endoscopic GEJ biopsies, and one gastroesophagectomy specimen. Patients’ average age was 53 years. There was no sex predilection (male to female ratio is 1.2:1).

For the 361 gastric and GEJ mucosal biopsies, the most common indications for endoscopy were epigastric pain, gastric ulcer, belching, and gastroesophageal reflux disease (Tables 1 and 2). For MSGC-positive GEJ specimens, the most commonly associated pathologic diagnosis was gastroesophageal reflux. Reflux was found in significantly greater percentage of MSGC-positive cases (73%, 11 of 15) than in the MSGC-negative cases (41%, 68 of 167; *p* value 0.03). The presence of MSGCs in the GEJ biopsies did not appear to be significantly associated with acute inflammation (*p* value 0.17), chronic inflammation (*p* value 0.99), intestinal metaplasia (*p* value 0.60), or pancreatic acinar cell metaplasia in the background mucosa (*p* value 0.22).

**Table 1:** Clinicopathologic Features of Gastroesophageal Specimens with Multinucleated Stromal Giant Cells

Case	Indication for Procedure	Pathology Diagnosis	Immunohistochemistry^a^	
			SMA	Desmin
1	Belching	Chronic inactive gastritis	+	+
2	GERD	CAI, REC	+/−	+/−
3	Alcohol abuse	RCG	+	+
4	Abdominal pain	CAI, PACM	+	+
5	Obesity S/P bypass	BE	+	+/−
6	Dysphagia	PACM, chronic inflammation	+	+/−
7	Esophagitis	BE, CAI, REC	+	+
8	Esophageal ulcer	ACG,RCG	+	+
9	BE, GERD	RCG	+	+
10	Abdominal pain	RCG	+	+
11	Obesity, heart burn	GERD	+	+/−
12	BE, GERD	REC, chronic inflammation	+	+
13	Epigastric pain	Active chronic gastritis, IM	+	+
14	BE with HGD	Rare AI	+	+
15	BE with LGD	CAI, REC, GERD	+	+
16	S/P resection for esophageal CA	Radiation atypia, REC, CAI	+/−	+
17	S/P resection for GEJ CA	Radiation atypia, BE with HGD	+	+
18	GERD, gastric CA	CAI, IM, luminal yeast/bacteria	+	+
19	Chronic gastritis, ulcer	CAI	+	+
20	GERD	PACM, chronic inflammation	+	+
21	Epigastric pain	RCG, chronic inflammation	+	+
22	Epigastric pain	No significant change	+	+

Abbreviations**:** *GERD* gastroesophageal reflux disease, *BE* Barrett’s esophagus, *HGD* high grade dysplasia, *LGD* low grade dysplasia, *CA* carcinoma, *GEJ* gastroesophageal junction, *REC* reactive epithelial changes, *CAI* chronic and acute inflammation, *RCG* reactive (chemical) gastropathy, *PACM* pancreatic acinar cell metaplasia, *IM* intestinal metaplasia, *S/P* status post

^a^The multinucleated stromal giant cells do not express cytokeratin AE1/3, chromogranin, CD68, CD117, and S100

**Table 2:** Summary of Clinicopathologic Features of Gastroesophageal Specimens with Multinucleated Stromal Giant Cells

Site (No.)	Common Clinical Symptoms	Other Pathologic Features	No. (%)
Gastroesophageal junction (15)	GERD; epigastric pain	GERD-type reactive epithelial changes	11 (73%)
		Chronic ± acute inflammation	8 (73%)
		Pancreatic acinar cell metaplasia	3 (20%)
		Intestinal metaplasia	2 (13%)
Stomach (7)	Epigastric pain, belching, gastric ulcer	Reactive (chemical) gastropathy	5 (71%)
		Chronic inflammation	4 (57%)
		Acute inflammation	2 (29%)
		Intestinal metaplasia	1 (14%)

Abbreviation: *GERD* gastroesophageal reflux disease

For MSGC-positive gastric biopsies, the most commonly associated pathologic diagnosis was reactive (chemical) gastropathy. Reactive (chemical) gastropathy was present in 71% (5 of 7) MSGC-positive gastric biopsies, in contrast to 34% (60 of 173) in MSGC-negative gastric biopsies. The association of reactive (chemical) gastropathy with MSGCs (*p* value 0.11) was not statistically significant at the 0.05 level, but there was an indication of potential association that may be detected with larger sample size. The presence of MSGCs in the gastric biopsies was not significantly associated with acute inflammation (*p* value 0.69), chronic inflammation (*p* value 0.99), or intestinal metaplasia (*p* value 0.92) in the background gastric mucosa.

**Fig. 1:**
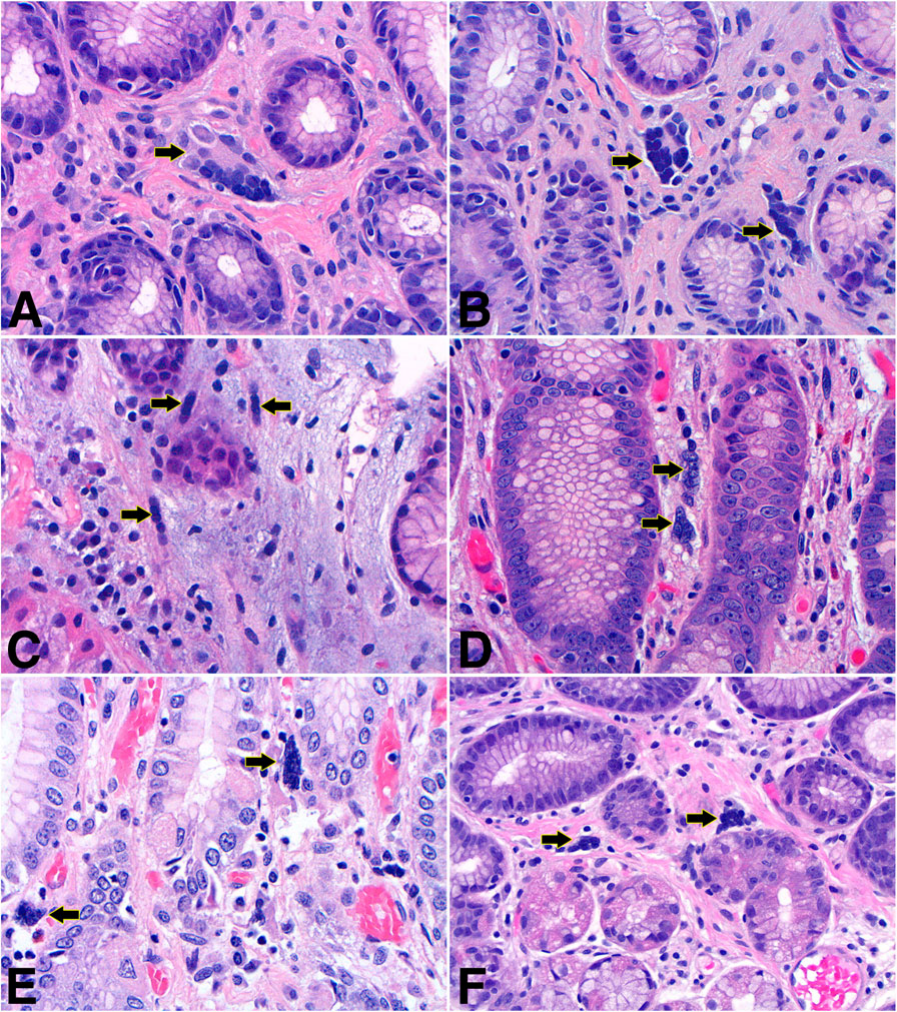
Histological features of multinucleated stromal giant cells. (**a** and **b**) Wreath-like nuclei in a patient with reactive gastropathy. (**c** and **d**) Caterpillar-like nuclei in two patients with chronic inactive gastritis. (**e** and **f**) Morular-like nuclei in a gastroesophageal resection of esophageal adenocarcinoma status post chemoradiation therapy (**e**) and a GEJ biopsy with gastroesophageal reflux disease (**f**). Hematoxylin and eosin stain, original magnification 600x

**Fig. 2:**
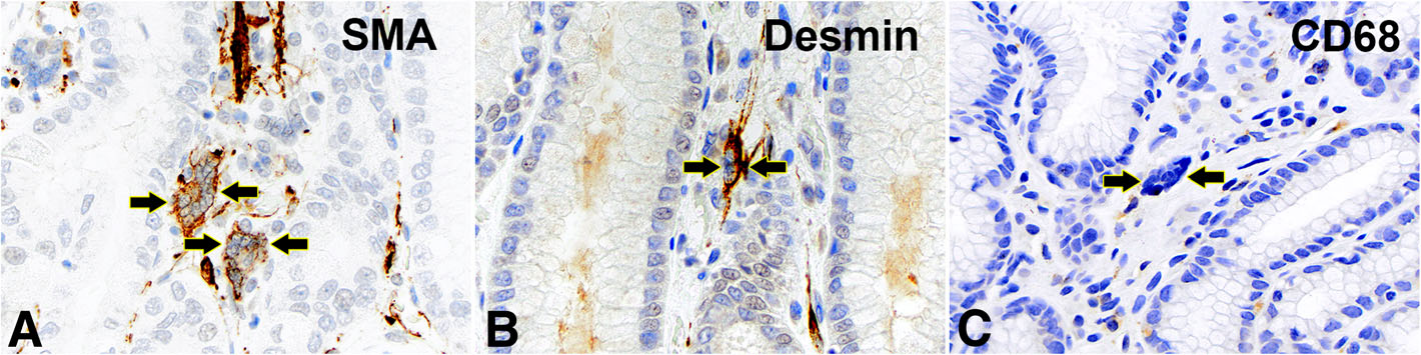
Immunohistochemical findings of multinucleated stromal giant cells. (**a**) Reactive for smooth muscle actin. (**b**) Reactive (variably) for Desmin. (**c**) Non-reactive for CD68. Original magnification 600x

MSGCs were located in the lamina propria of the gastric/GEJ mucosa, with an accentuation in the subepithelial zone. Most cases (77%) had only 1 or 2 MSGCs per 10 high power fields, while occasional cases had slightly more MSGCs (up to 5 per 10 high power fields). The cells were stellate or epithelioid in shape with little cytoplasm and a median number of 5 nuclei (ranges from 3 to 16 nuclei). Due to the scanty cytoplasm, MSGCs resembled “a bag of nuclei”. The nuclei were hyperchromatic, and touching/overlapping. They were often arranged into one of the three configurations: “wreath” – circular arrangement of the nuclei in the periphery of the cytoplasm (Fig. 1a,b, arrows), “caterpillar” – linear arrangement of the nuclei (Fig. 1 c,d, arrows), or “morular” – random arrangement of the nuclei in a cluster (Fig. 1e,f, arrows). On immunohistochemistry, MSGCs expressed SMA (Fig. 2a, arrows) and variably expressed desmin (Fig. 2b, arrows). MSGCs were negative for CD68 (Fig. 2c, arrows), cytokeratin AE1/3, S100, chromogranin, and CD117.

## Discussion

To the best of our knowledge, this is the first study to evaluate and characterize MSGCs in the upper gastrointestinal tract. Given that MSGCs are most commonly seen in the setting of reflux and chemical/reactive gastropathy, they are likely the sequelae of a reactive/regenerative process. The immunohistochemical reactivity for SMA and variable desmin is consistent with smooth muscle/myofibroblast differentiation. The non-reactivity for cytokeratin AE1/3, CD68, S100, chromogranin, and CD117, argues against the following differentiation respectively - epithelial, histiocytic, schwannian, neuroendocrine, or interstitial cell of Cajal. MSGCs sometimes appear to be surrounded by stromal collagen (Fig. 1a,b,f) or in continuation with the upward stranding muscularis mucosae (Fig. 1c), supporting possible myofibroblastic/smooth muscle origin. In the setting of reflux and reactive (chemical) gastropathy, there is a well-known stromal fibromuscular change. These observations together with the immunoprofile suggest MSGCs most likely represent regenerative cells from the upward stranding muscularis mucosae or stromal myofibroblasts. This theory is in keeping with analogous studies on colonic MSGCs [3]. In a study of MSGCs in the lower gastrointestinal tract [3], MSGCs were reported to exist in 23% of biopsies from both normal and abnormal colonic mucosa. The abnormal mucosa included tubular adenoma, focal active colitis, hyperplastic polyp, etc. MSGCs were not identified in the 30 rectal cases in their cohort. We have also observed MSGCs in ischemic colitis and CMV colitis cases (data not shown).

At times MSGCs may appear atypical and raise concern for malignancy based on their large size, hyperchromatic nuclei, and background stromal changes. Reassuring features of benignity include low cellularity, an absence of mitoses, and cytokeratin negativity. In addition, background reflux-type mucosal changes in a GEJ specimen and erosion/ulcer/reactive (chemical) gastropathy changes in a gastric or esophageal biopsy are also reassuring.

**Fig. 3:**
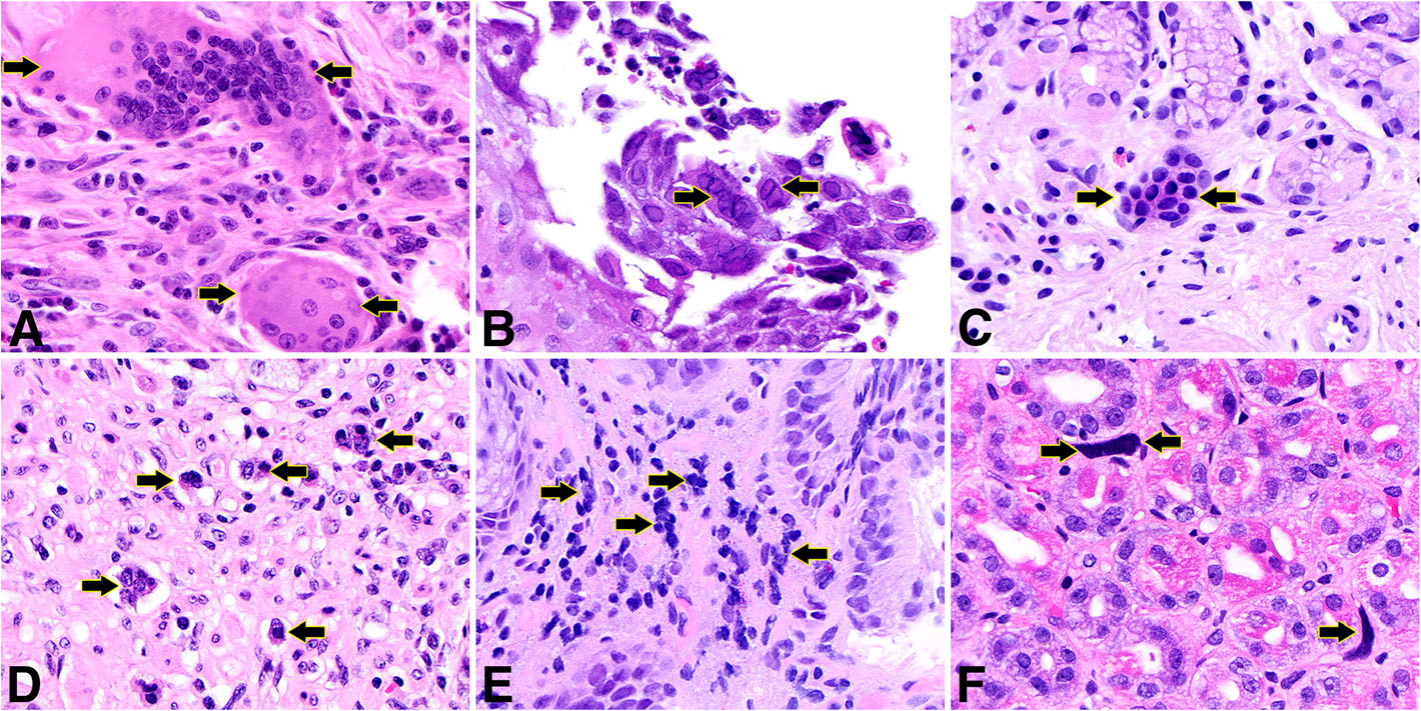
Other multinucleated cells and mimics in the gastroesophageal mucosa. (**a**) Foreign-body-type multinucleated histiocyte, stomach. (**b**) Herpes Simplex viral cytopathic effect, esophagus. (**c**) Tangentially sectioned pit epithelium, stomach. (**d**) Tight clusters of neuroendocrine cells (neuroendocrine nests post chemoradiation therapy), gastroesophageal junction. (**e**) Cluster of crushed inflammatory cells, gastroesophageal junction. (**f**) Benign megakaryocytes in the setting of myelofibrosis, stomach. Hematoxylin and eosin stain, original magnification 600x

Other types of multinucleated cells and mimickers are sometimes found in the upper gastrointestinal tract. Multinucleated histiocytes are seen in granulomas of various etiologies and foreign-body giant cell reaction. However, these histiocytic multinucleated cells are morphologically distinct from MSGCs in that they contain abundant eosinophilic cytoplasm (Fig. 3a). Herpes simplex virus (HSV)-infected cells feature multinucleated epithelial cells with ground glass nuclear inclusions (Fig. 3b). Unlike MSGCs, however, HSV–infected nuclei contain smudged chromatin and nuclear molding. Tangentially-sectioned glandular epithelium may look like a multinucleated cell from low-power; however, these nuclei are more regularly placed with a honeycomb appearance (Fig. 3c). Be aware that tight clusters of small-sized cells in the esophagogastric mucosa, such as neuroendocrine nests post chemoradiation (Fig. 3d), or collection of crushed inflammatory cells (Fig. 3e) can mimic MSGCs. Benign megakaryocytes in the gastric mucosa of patients with myelofibrosis can also be in the differential diagnosis of MSGCs (Fig. 3f); however, the nuclei of megakaryocytes are much larger than MSGCs. A familiarity with the usual appearance of MSGC is reassuring.

## Conclusions

MSGCs in the gastric and gastroesophageal junctional mucosa show smooth muscle/myofibroblast differentiation, and could represent regenerative cells from muscularis mucosae or stromal myofibroblasts, as part of reactive stromal changes that are often associated with gastroesophageal reflux and reactive (chemical) gastropathy.

## Data Availability

The datasets used and/or analyzed for the study are available from the corresponding author on reasonable request.

## Abbreviations

BE: Barrett esophagus
CA: Carcinoma
CAI: Chronic and acute inflammation
GEJ: Gastroesophageal junction
GERD: Gastroesophageal reflux disease
HGD: High grade dysplasia
HSV: Herpes simplex virus
IM: Intestinal metaplasia
LGD: Low grade dysplasia
MSGCs: Multinucleated stromal giant cells
PACM: Pancreatic acinar cell metaplasia
RCG: Reactive (chemical) gastropathy
REC: Reactive epithelial change
S/P: Status post
